# The molecular mechanisms underlying arecoline-induced cardiac fibrosis in rats

**DOI:** 10.1515/biol-2021-0116

**Published:** 2021-11-02

**Authors:** Chang-Wen Ku, Cecilia Hsuan Day, Hsiu-Chung Ou, Tsung-Jung Ho, Ray-Jade Chen, Velmurugan Bharath Kumar, Wen-Yuan Lin, Chih-Yang Huang

**Affiliations:** Department of Chinese Medicine, Hualien Tzu Chi Hospital, Buddhist Tzu Chi Medical Foundation, Hualien, Taiwan; Integration Center of Traditional Chinese and Modern Medicine, Hualien Tzu Chi Hospital, Buddhist Tzu Chi Medical Foundation, Hualien, Taiwan; Department of Nursing, MeiHo University, Pingtung, Taiwan; Department of Physical Therapy, College of Medical and Health Science, Asia University, Taichung, Taiwan; School of Post-Baccalaureate Chinese Medicine, College of Medicine, Tzu Chi University, Hualien, Taiwan; Department of Surgery, School of Medicine, College of Medicine, Taipei Medical University, Taipei 11031, Taiwan; Department of Biotechnology, Asia University, Taichung, Taiwan; The Department of Family Medicine, China Medical University Hospital, Taichung, Taiwan; Department of Medical Laboratory Science and Biotechnology, Asia University, Taichung, Taiwan; Cardiovascular and Mitochondrial Related Disease Research Center, Hualien Tzu Chi Hospital, Buddhist Tzu Chi Medical Foundation, Hualien, Taiwan; Department of Medical Research, China Medical University Hospital, Taichung, Taiwan; Center of General Education, Tzu Chi University of Science and Technology, Hualien, Taiwan; Graduate Institute of Biomedical Sciences, China Medical University Hospital, Taichung 404, Taiwan

**Keywords:** arecoline, cardiac fibrosis, TGF-β, MMP9, Smad

## Abstract

The areca nut is one of the most commonly consumed psychoactive substances worldwide, with an estimated consumption by approximately 10% of the world’s population, especially in some regions of South Asia, East Africa, and the tropical Pacific. Arecoline, the major areca nut alkaloid, has been classified as carcinogenic to humans as it adversely affects various organs, including the brain, heart, lungs, gastrointestinal tract, and reproductive organs. Earlier studies have established a link between areca nut chewing and cardiac arrhythmias, and yet research pertaining to the mechanisms underlying cardiotoxicity caused by arecoline is still preliminary. The main purpose of this study is to test the hypothesis that arecoline causes cardiac fibrosis through transforming growth factor-β (TGF-β)/Smad-mediated signaling pathways. Male Wistar rats were injected intraperitoneally with low (5 mg/kg/day) or high (50 mg/kg/day) doses of arecoline for 3 weeks. Results from Masson’s trichrome staining indicated that arecoline could induce cardiac fibrosis through collagen accumulation. Western blot analysis showed that TGF-β and p-Smad2/3 protein expression levels were markedly higher in the arecoline-injected rat hearts than in those of the control rats. Moreover, arecoline upregulated other fibrotic-related proteins, including SP1-mediated connective tissue growth factor expression. Tissue-type plasminogen activator and its inhibitor, plasminogen activator inhibitor, and matrix metalloproteinase (MMP) 9 were upregulated, and the inhibitor of MMP9 was downregulated. This study provides novel insight into the molecular mechanisms underlying arecoline-induced cardiac fibrosis. Taken together, the areca nut is a harmful substance, and the detrimental effects of arecoline on the heart are similar to that caused by oral submucous fibrosis.

## 1 Introduction

**Figure 1:**
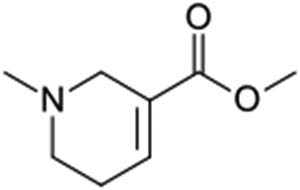
Chemical structure of arecoline, the principal alkaloid in the areca nut.

More than 600 million people are regular consumers of areca nuts, especially people in Asian populations [1]. Following nicotine, ethanol, and caffeine, the areca nut is considered the fourth most commonly abused substances by people [2]. Numerous psychiatric studies have shown that chronic areca nut chewers are prone to developing drug dependence, and their presenting symptoms meet the criteria for a substance use disorder of the *Diagnostic and Statistical Manual of Mental Disorders*, Fifth Edition [3]. In some regions of the tropical Pacific, East Africa, and South Asia, the areca nut is served as a food substance in social gatherings and religious festivities for people with lower socioeconomic status [1]. Chewing the areca nut usually slightly elevates the body temperature and causes people to feel energetic, euphoric, and vigilant. Several epidemiological studies have demonstrated that chronic areca nut chewers can develop systematic diseases in various organs, including the brain, heart, lungs, gastrointestinal tract, reproductive organs, and immune system [4]. Consuming areca nuts has been demonstrated to cause hyperlipidemia, vasospasm, and cardiac arrhythmias, which can subsequently lead to an increased risk of myocardial ischemia [5]. Arecoline, *N*-methyl-1,2,5,6-tetrahydropyridine-3-carboxylic acid methyl ester (Figure 1), the principle alkaloid in the areca nut, has been classified as carcinogenic to humans [6]. Arecoline-induced collagen deposition through multisignaling pathways has been postulated as the primary causative event for increased collagen production in oral submucous fibrosis [7]. However, information regarding the cardiac dysfunction caused by arecoline is relatively limited.

Fibrosis is characterized by scarring and tissue hardening and is typically caused by excessive extracellular matrix (ECM) protein deposition by myofibroblasts while responding to chronic inflammation [8,9]. A variety of noxious stimuli, including toxins, infectious pathogens, autoimmune reactions, and mechanical stress, are capable of causing fibrotic cellular responses [10]. Fibrosis can occur in almost all organs, including the heart, lungs, liver, and kidneys [11]. Upon tissue damage, myofibroblasts, which are derived from various sources such as resident fibroblasts, circulating fibrocytes, and mesenchymal cells, undergo a wound healing process to restore tissue integrity and promote the replacement of parenchymal cells through the remodeling of the extracellular environment. This pro-fibrotic program is then typically terminated once the tissue healing process has been completed. However, persistent insults or tissue damage can cause dysregulation of the process, resulting in excessive deposition of ECM proteins and reinforced myofibroblast activities [12].

Transforming growth factor-β (TGF-β), the best-known fibrogenic growth factor, is the main regulator of tissue growth, regeneration, remodeling, and fibrosis [13]. The TGF-β family is composed of three subtypes, namely TGF-β1, TGF-β2, and TGF-β3, where TGF-β1 is considered as the major driver of human fibrotic pathologies [14]. Activation of TGF-β1 signaling has been postulated as the main causative event for increased collagen production in oral submucous fibrosis [15]. Recently, it has been shown that the expression of TGF-β is increased during fibrosis, and its downstream mediator Smad2/3 is involved in cardiac fibrosis [16]. Activation of the Smad3 signaling pathway has been reported to play an important role in modulating TGF-β-induced ECM protein synthesis [17]. TGF-β exerts matrix-preserving actions via modulating the activity of matrix metalloproteinases (MMPs) and their inhibitor, tissue inhibitor of metalloproteinases (TIMPs) [18], as well as via regulating the expression of plasminogen activator (PA) and its inhibitors, including PA inhibitor (PAI) [19].

Connective tissue growth factor (CTGF), a cysteine-rich 36–38 kDa secreted protein, is a matricellular protein involved in regulating cell survival, proliferation, adhesion, migration, and ECM production [20]. In addition, TGF-β can induce the *Ctgf* gene expression in various cell types, especially in fibrotic lesions, and the promoter of the *Ctgf* gene includes a TGF-β1 response element [21]. Elevated CTGF expression was found in infarcted hearts [22], as well as in cardiac samples obtained from patients who suffer from heart failure [23]. Moreover, the CTGF-stained areas corresponded to the myocardial fibrosis area [24]. Conversely, some of the pro-fibrotic effects of TGF-β have been reported to be mediated via upregulation of its downstream effector CTGF. TGF-β induces the expression of CTGF via a functional Smad3 binding site in the *Ctgf* gene promoter, which subsequently promotes the synthesis of collagen and the differentiation of myofibroblasts [25]. Furthermore, other studies of *in vivo* models have concluded that CTGF potentiates fibrogenic actions via regulating TGF-β. However, inhibition of CTGF enhances cardiac repair and limits fibrosis after myocardial infarction [26].

The behavior of chewing areca nuts habitually has been found to cause cardiotoxicity, including coronary artery disease [27], heart failure, and premature ventricular contractions [28]. However, there is a lack of research investigating the effect of arecoline on cardiac fibrosis. As such, we aimed to determine whether arecoline can cause cardiac fibrosis in an animal model and, if so, whether the underlying mechanisms involve the TGF-β/Smad signaling pathway.

## 2 Materials and methods

### 2.1 Antibodies and reagents

All chemicals and reagents were purchased from Sigma-Aldrich (St. Louis, MO). Primary antibodies used in the study include p-Smad2/3 (Cell Signaling Technology, Danvers, MA), TGF-β, MMP9, TIMP-2, CTGF, tissue-type PA (tPA), and β-actin (Santa Cruz Biotechnology, Dallas, TX). All secondary antibodies (anti-rabbit, anti-mouse, and anti-goat horseradish peroxidase-conjugated antibodies) were purchased from Santa Cruz Biotechnology (Dallas, TX).

### 2.2 Animal procedure

**Figure 2:**
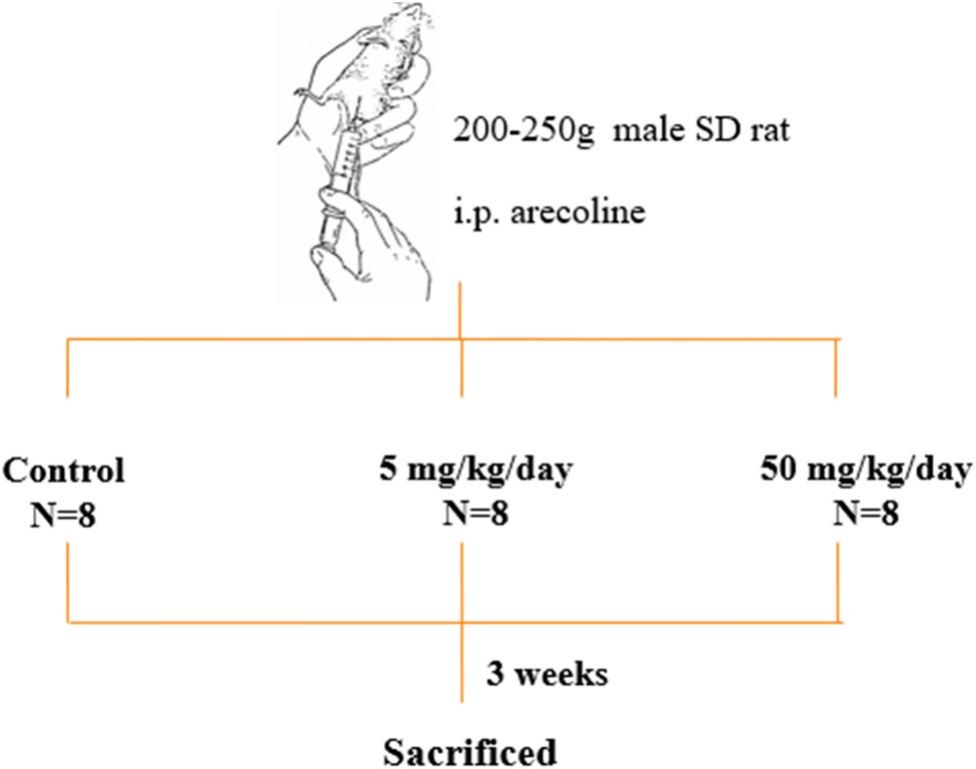
Administration of arecoline to animals.


Sprague-Dawley (SD) rats were purchased from BioLASCO Co., Ltd. (Taipei, Taiwan). The rats were provided with standard laboratory chow and water *ad libitum*. Eight-week-old male SD rats were individually housed in a temperature-controlled room at 25 ± 2°C with a 12 h dark–light cycle. After a 4-week acclimation period, the rats were divided into three groups, with eight animals in each group: control group, arecoline low-dosage group (5 mg/kg/day), and arecoline high-dosage group (50 mg/kg/day). Phosphate-buffered saline was used as the medium in all groups. Rats were given arecoline with low dosage or high dosage via intraperitoneal (i.p.) injection every day for 3 weeks (Figure 2). After the treatment, the animals were weighed, anesthetized via isoflurane exposure, and sacrificed via cervical decapitation, and the heart tissue was collected and stored at −80°C until further Western blot analysis. For histological analysis, the collected whole heart tissues from each group were immersed in 10% formalin with gently shaking for 48 h and then dehydrated by consecutive immersion in alcohols and fixed with paraffin wax.

**Ethical approval:** The research related to animal use has been complied with all the relevant national regulations and institutional policies for the care and use of animals and was approved by the Animal Research Committee of China Medical University, Taichung, Taiwan and the Animal Care and Use Committee at China Medical University (approval number 100-4-B).

### 2.3 Masson’s trichrome (MT) staining

Two-micron thick paraffin sections from the hearts of rats in each group were cut from paraffin-embedded tissue blocks. Whole-heart cross sections, derived from serial sectioning, were deparaffinized by immersion in xylene and then rehydrated. The samples were then exposed to MT stain (ScyTek Laboratories, Inc., Logan, UT) and histologically evaluated for fibrotic changes in the heart sections. Images of the samples were obtained microscopically (Zeiss Axiophot, Oberkochen, Deutschland, Germany) under 400× magnification. Semiquantitative scoring (0 to 4) of trichrome sections was assigned in a blinded manner independently by two researchers (Dr Ku and Dr Ou).

### 2.4 Tissue protein extraction

The left ventricle tissues were collected and homogenized using lysis buffer (20 mM Tris, 2 mM ethylenediaminetetraacetic acid, 50 mM β-mercaptoethanol, 10% glycerol, protease inhibitor, and phosphatase inhibitor, pH 7.4). The homogenates were centrifuged at 12,000×*g* for 40 min, and the supernatant was collected and stored for further analysis [29].

### 2.5 Western blotting

Western blot analysis for protein expression was similar to previously described, with slight modifications [30]. The protein concentration of the tissue was analyzed by Lowry’s protein assay [31]. Proteins (40 μg/lane) were separated by 10–15% gradient sodium dodecyl sulphate–polyacrylamide gel electrophoresis with a mini gel apparatus at 75 V for 3 h and then transferred to a polyvinylidene fluoride membrane and blocked with 5% nonfat dry milk in tris-buffered saline buffer. Primary antibodies (TGF-β1, p-Smad2/3, SP1, CTGF, tPA, PAI-1, MMP9, TIMP, and β-actin) were diluted to 1:500 and added to hybridize to the membrane overnight at 4°C. Then, the membranes were washed with tris buffered saline buffer with Tween 20 buffer for 30 min, and the secondary antibody solution (1:5,000 dilution) was added and allowed to incubate for 1 h. The proteins were visualized using a enhanced chemiluminescence Western blotting reagent (Millipore, Burlington, MA) in Fujifilm LAS-3000 (GE Healthcare Life Sciences, Marlborough, MA). The intensity of the protein bands was quantified using ImageJ software https://imagej.nih.gov/ij/download.html, and the densitometric data were normalized using β-actin as an internal control.

### 2.6 Statistical analysis

Statistical analysis was performed with GraphPad Prism software, version 5.0 (Graph-Pad Software, San Diego, CA, USA). All data are expressed as mean ± standard error of the mean. Comparative analysis between groups was conducted using one-way analysis of variance. Significance between the individual means was determined by Tukey’s test. A *P* value of less than 0.05 was considered significant.

## 3 Results

### 3.1 Arecoline induces cardiac fibrosis

**Figure 3:**
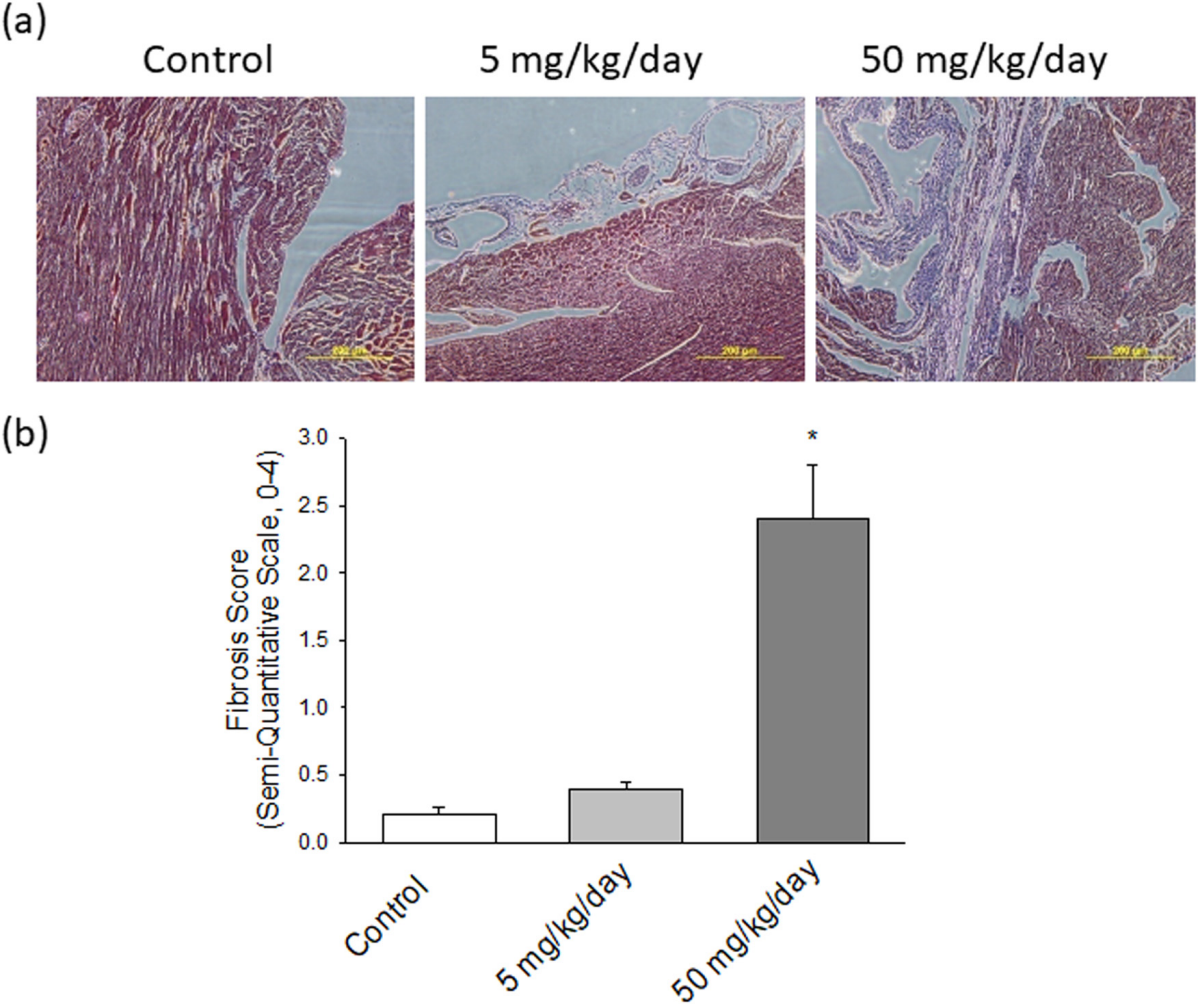
(a) Pathological changes in the left ventricles of experimental rats. MT staining of cardiac tissues from control, low-dosage arecoline, and high-dosage arecoline rats. Collagen accumulation is shown in blue. The myocardial architecture images were magnified at ×400. The scale bar is 200 µm. (b) Semiquantitative grade morphormetric fibrosis scoring for trichrome slides of left ventricular cardiac section. * Significant difference (*P* < 0.05).

To investigate the role of arecoline-affected signaling molecules on cardiac fibrosis, MT staining was implemented to analyze the collagen accumulation in ventricular tissue. The collagen fibers are stained blue, the nuclei are stained black, and the background is stained red. Compared to control rats (Figure 3a, left panel), heart damage due to slight collagen accumulation in rats subjected to low-dosage arecoline treatment was observed (Figure 3a, middle panel); however, the content of accumulated collagen was significantly higher in high-dosage arecoline treatment (Figure 3a, right panel). The accumulation of cardiac collagen fibers clearly indicates the fibrotic injury in the arecoline treatment group.

### 3.2 Arecoline induces TGF-β1/Smad2/3 signaling

**Figure 4:**
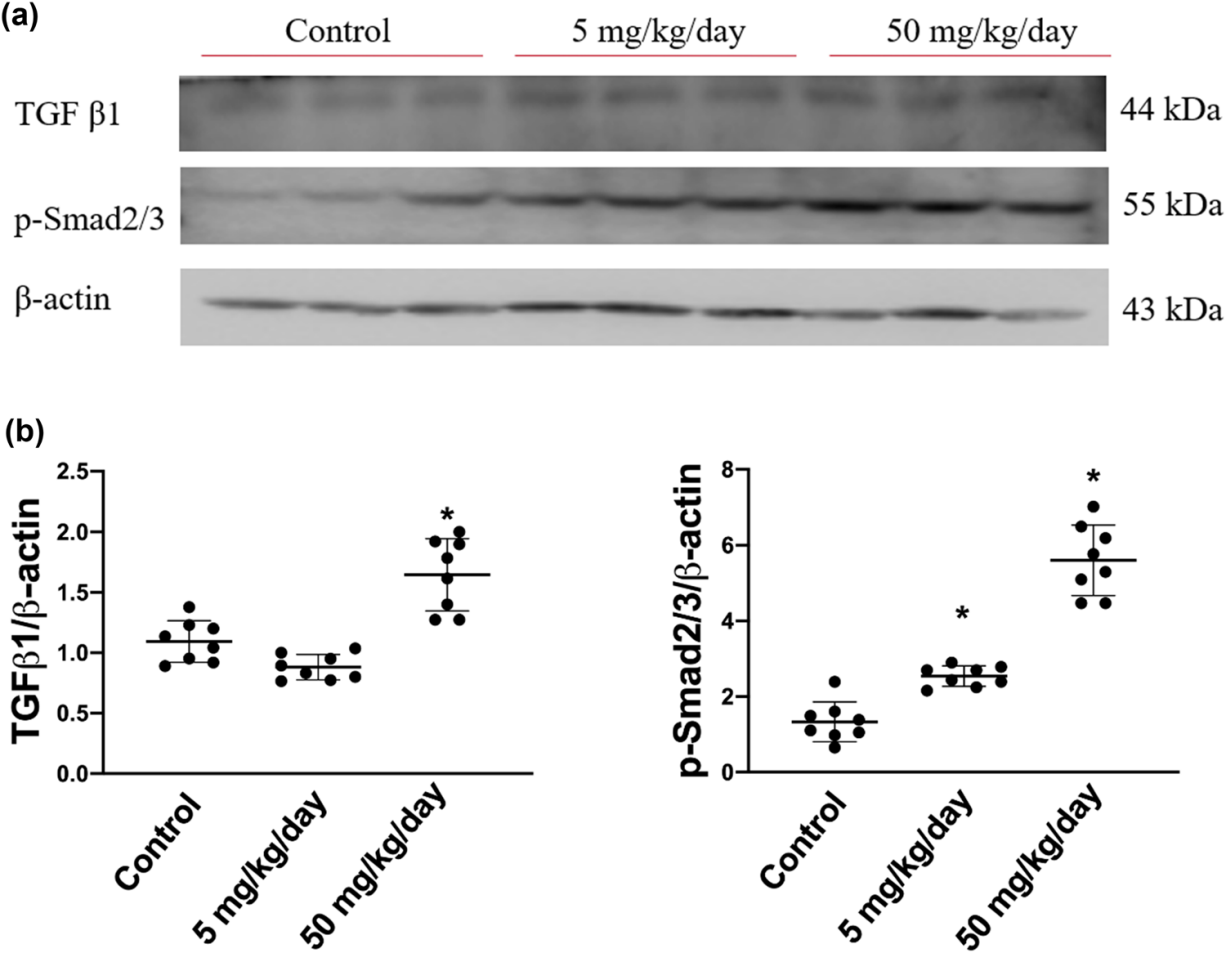
TGF-β1 and phosphorylated Smad2/3 protein expression levels in arecoline-treated rats. (a) TGF-β1 and p-Smad2/3 protein expression levels were examined by Western blot analysis of left ventricular samples from control, low-dosage, and high-dosage arecoline-treated rats. (b) Data were quantified densitometrically and expressed as mean ± SEM. Protein expression was normalized to β-actin expression. *Significant difference (*P* < 0.05).

To investigate the pro-fibrotic protein expression signaling pathways affected by arecoline, we first assessed the levels of molecules involved in the progress of cardiac remodeling, namely TGF-β1 and p-Smad2/3, in control, low-dose arecoline, and high-dose arecoline rats. The TGF-β1 expression was slightly lower in the low-dose arecoline rats compared to the control rats, and it exhibited an approximately 1.7-fold increase in high-dose arecoline rats compared to the control rats (Figure 4). In terms of p-Smad2/3, the downstream molecule of TGF-β1, the expression levels were increased by 2- and 4.8-fold in low-dose and high-dose arecoline rats compared to control rats, respectively. The results suggest that TGF-β1 signaling is involved in the cardiac fibrosis caused by arecoline, especially in the high-dose group. Notably, the expression levels of TGF-β1 in low-dose group rats are slightly decreased compared to that of the control group.

### 3.3 Arecoline induces SP1-mediated CTGF expression

**Figure 5:**
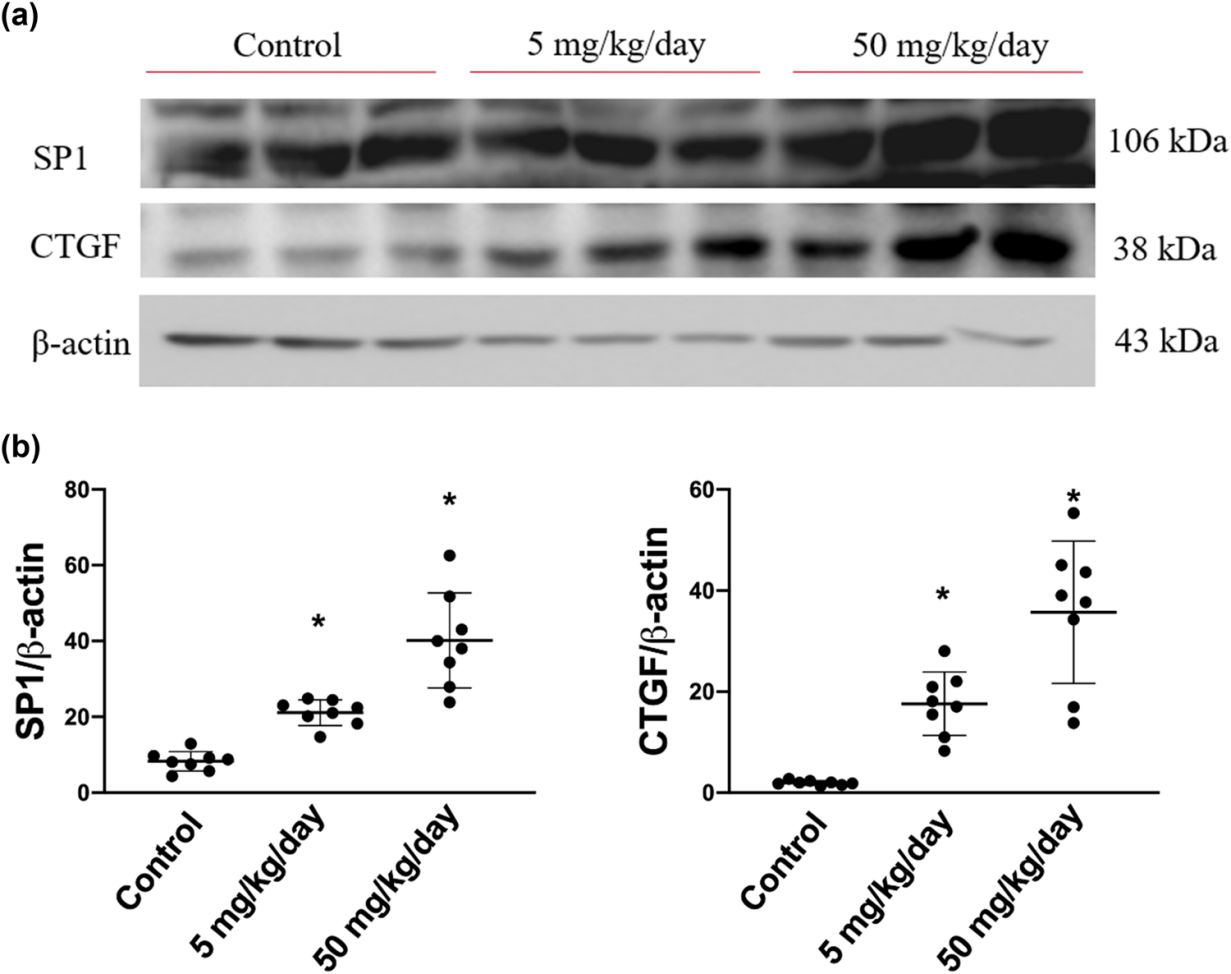
SP1 and CTGF protein expression levels in arecoline-treated rats. (a) SP1 and CTGF protein expression levels were examined by Western blot analysis of left ventricular samples from control, low-dosage, and high-dosage arecoline-treated rats. (b) Data were quantified densitometrically and expressed as mean ± SEM. Protein expression was normalized to β-actin expression. *Significant difference (*P* < 0.05).

Based on the abovementioned findings, we further determined the protein expression levels of CTGF and its transcription factor SP1, which are both regulated by the TGF-β1-activated p-Smad2/3 pathway. As expected, the expression levels of SP1 were dose dependently increased in arecoline-treated rats compared to the control rats (all *P* < 0.05). Similarly, the protein levels of CTGF are significantly elevated in both low- and high-dose arecoline-treated groups (Figure 5). The results confirm our hypothesis that the signaling pathway of TGF-β1/Smad2/3/SP1/CTGF is involved in, at least in part, the cardiac fibrosis caused by arecoline.

### 3.4 Arecoline induces PAI/tPA

**Figure 6:**
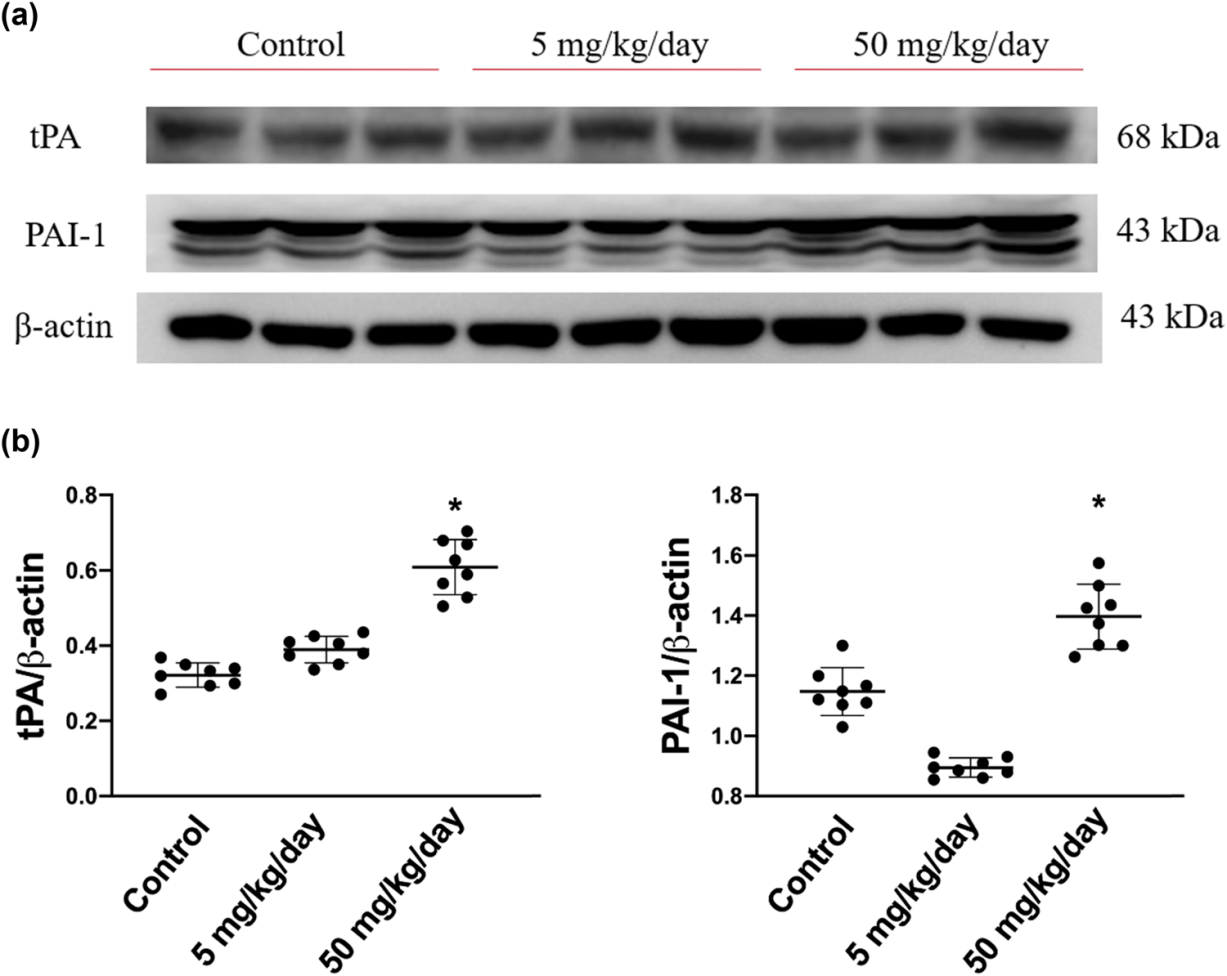
tPA and its inhibitor, PAI protein expression levels in arecoline-treated rats. (a) PAI and tPA protein expression levels were examined by Western blot analysis of left ventricular samples from control, low-dosage, and high-dosage arecoline-treated rats. (b) Data were quantified densitometrically and expressed as mean ± SEM. Protein expression was normalized to β-actin expression. *Significant difference (*P* < 0.05).

Subsequently, we sought to determine whether the expression levels of PAI/tPA are involved by arecoline-induced cardiac fibrosis. As shown in Figure 6, the expression levels of PAI were significantly increased in rats in the high-dosage group compared to rats in the low-dosage and the control groups. Similarly, the expression levels of tPA were markedly elevated only in the high-dose group (Figure 6). Notably, the expression levels of PAI-1 in low-dosage group rats are slightly reduced compared to those in the control group. Based on the results, we suggest that arecoline-induced cardiac fibrosis through tPA/PAI-1 regulated the signaling pathway, especially in high-dosage group rats.

### 3.5 Arecoline induces compensatory inhibition of TIMP2/MMP9 signaling

**Figure 7:**
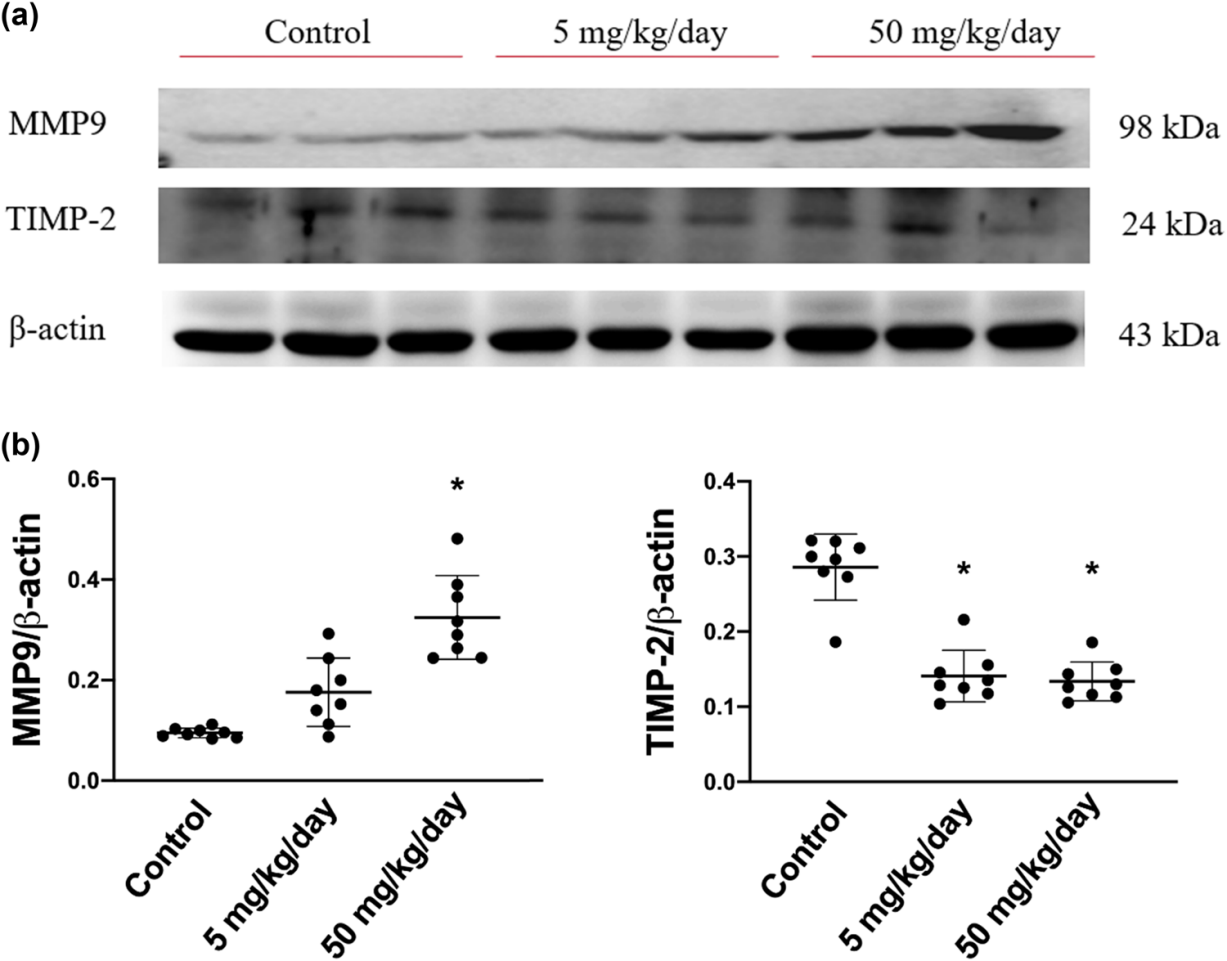
MMP9 and its inhibitor, TIMP2, protein expression levels in arecoline-treated rats. (a) MMP9 and TIMP2 protein expression levels were examined by Western blot analysis of left ventricular samples from control, low-dosage, and high-dosage arecoline-treated rats. (b) Data were quantified densitometrically and expressed as mean ± SEM. Protein expression was normalized to β-actin expression. *Significant difference (*P* < 0.05).

We subsequently measured the expression levels of MMP9 and its inhibitor, TIMP2. We found that the expression levels of MMP9 were markedly elevated in high-dosage group rats, whereas the expression level of TIMP2 was significantly reduced in both low-dosage and high-dosage arecoline-treated groups (Figure 7). The results are consistent with earlier studies that MMP-9 influences the metabolism of collagen and promotes the fibrosis of the myocardium. Accordingly, we suggested that MMP-9 participates in arecoline-induced cardiac fibrosis, especially in high-dosage group rats.

## 4 Discussion

**Figure 8:**
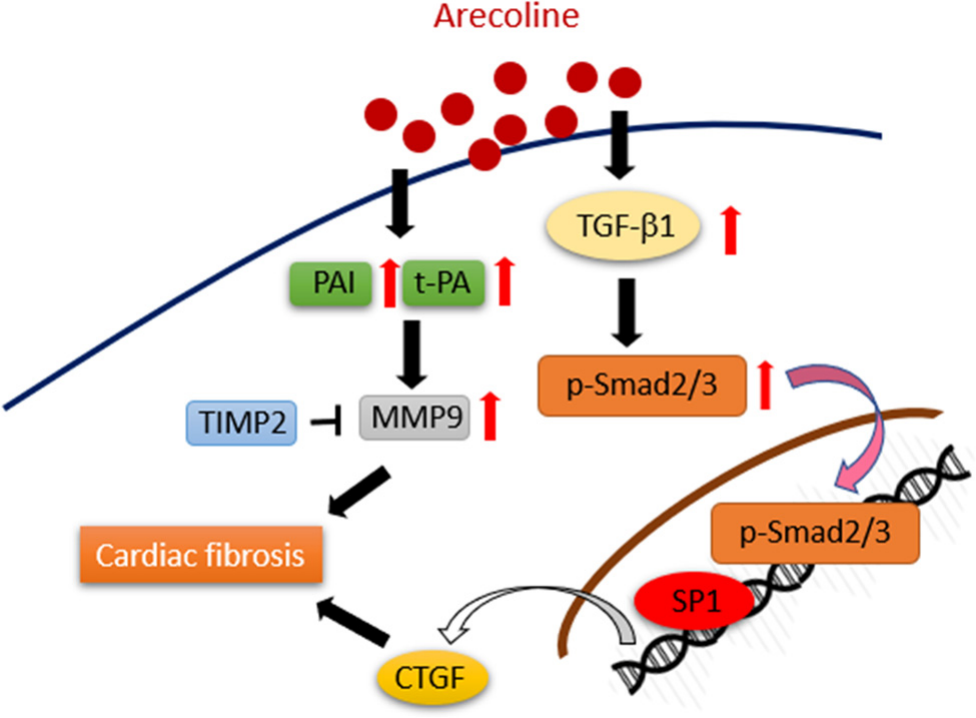
Schematic showing how arecoline induces cardiac fibrosis through upregulation of the TGF-β1/Smad2/3/CTGF as well as tPA/MMP9 signaling pathways.

To the best of our knowledge, this is the first study to detail the molecular mechanisms through which arecoline induces cardiac fibrosis. In accordance with earlier research that found that arecoline induces oral submucous fibrosis through activation of the TGF-β1/Smad signaling pathway, our data provided show that the i.p. administration of arecoline significantly increased the protein expression levels of fibrotic modulators, including TGF-β1, p-Smad2/3, SP1, and CTGF. Furthermore, ECM remodeling proteins, including tPA and MMP9, were upregulated, and TIMP2 was downregulated during arecoline-induced cardiac fibrosis (Figure 8).

The molecular pathogenesis of arecoline on the oral cavity, which is the primary exposure site of areca nut chewing, has long been explored. Several arecoline-induced pro-fibrotic factors are accompanied by a reduced intracellular thiol content, and this impairment can be reversed in the presence of antioxidants [32]. Indeed, reactive oxygen species (ROS) generation by arecoline is considered an upstream cause of the various arecoline-driven effects that initiate oral submucous fibrosis [33]. Interaction in a reciprocal manner can be observed between ROS and TGF-β1. For instance, arecoline-induced ROS activates diverse downstream signaling molecules, including TGF-β1, which is followed by ECM deposition, and TGF-β1 reversely increases ROS production coupled with suppression of the antioxidative enzymes [33].

Cardiac fibroblasts play a significant role in the homeostasis of the ECM in cardiac tissue, and they have recently been identified as inflammatory supporter cells. TGF-β1 is a critical regulator of tissue growth, regeneration, remodeling, and fibrosis. TGF-β1 in the injured heart is expressed by resident macrophages and cardiac fibroblasts [34]. Through the activation of the downstream Smad pathway, TGF-β1 induces cardiac fibroblast to myofibroblast differentiation and collagen deposition [35]. Although the TGF-β superfamily is considered the main activator of Smad signaling, accumulating evidence suggests that Smad activation may also be attributed to TGF-β-independent mechanisms in certain cell types [14]. In this study, i.p. administration of arecoline at 5 mg/kg/day did not cause an increase of TGF-β1, and yet it did lead to an increase in p-Smad2/3, suggesting that Smad activation in arecoline-induced fibrotic cardiac conditions is due to both TGF-β-dependent and -independent pathways. In addition, a recent study published by our research group demonstrated that phosphor-Akt, a survival signaling molecule, was tended to increase in low-dosage arecoline-treated rats compared to the control group and high-dosage group rats. The results inferred that the myocardial tissue might still be protected by the survival mechanism under the lower dosage of arecoline but not at high dosage [36]. Consistently, results from this study also showed that the expression levels of TGF-β1 were slightly lower than those in control group rats.

CTGF induced the proliferation of fibroblasts, promoted the transformation from cardiac fibroblast to myofibroblasts, and increased ECM production. Earlier research has indicated that in addition to TGF-β stimulated Smads, several signaling molecules, such tumour necrosis factor α, epidermal growth factor, and wingless/integrated, are involved in the induction of CTGF in normal fibroblasts [37]. This study demonstrated that while arecoline did not change the expression levels of TGF-β1 at a low dose, there was still an increase in CTGF protein expression. This suggests that arecoline may have induced CTGF expression through TGF-β-dependent and -independent pathways.

PAs and their inhibitors, PAIs, play crucial roles in the balance of proteolytic and antiproteolytic enzymatic cascades that regulate ECM turnover. Fibrotic lesions occur when normal control of the balance becomes compromised, which leads to excessive deposition of ECM in the tissues. Consistent with a prior study that showed that both tPA and PAI are increased in areca nut chewer fibroblasts compared to control individual fibroblasts [38], our results indicated that the protein expression levels of tPA and PAI were significantly higher in rats that received a high dose of arecoline than in rats from the low-dose and the control groups. Furthermore, it has been reported that increased tPA has detrimental consequences by inducing MMP9 expression [39]. We found that MMP9 was upregulated, and its inhibitor TIMP2 was downregulated in a dose-dependent manner during arecoline-induced cardiac fibrosis.

In addition to the well-known pathogenic mechanisms underlying oral submucous fibrosis caused by arecoline, the adverse effects of areca nut in other organs have been discovered. For instance, habitual betel quid chewing caused hepatocellular carcinoma complicating cirrhosis [40], and arecoline-induced growth arrest and p21WAF1 expression are dependent on p53 in rat hepatocytes [41]. Betel nut chewing causes bronchoconstriction [42]. Arecoline causes neurotoxicity through enhancement of oxidative stress and suppression of the antioxidant protective system [43]. Several studies have been reported that betel nut chewing induced cardiac dysrhythmias [28,44]. However, the molecular mechanisms in which arecoline-induced cardiac dysfunction is needed to be further elucidated.

Areca nuts have been classified as a Class I carcinogen by IARC (IARC working Group on the Evaluation of Carcinogenic Risks to Human, 2004) [45]. Arecoline is an important alkaloid that can be detected in the blood plasma, hair samples, and breast milk of areca nut consumers [46]. The lower dose, 5 mg/kg/day, used in our study has been reported in an earlier pharmacokinetic study that the peak concentrations of 1142 ± 554 and 923 ± 368 ng/mL were measured in plasma after 1 min in 3- and 24-month-old rats, respectively. Thereafter, arecoline demonstrated a monophasic disappearance with *t*_l/2_ values of 5.8 and 3.5 min [47]. As such, the concentrations of arecoline in the plasma after i.p. administration at the dose of 5 mg/kg/day were likely depleted within an hour. Accordingly, we assumed that the expression levels of TGF-β1 in rats that received the low-dosage arecoline remained unchanged or slightly lower than the control is attributed to the short half-life of arecoline in the plasma. Another human pharmacokinetics study reported that the concentration of arecoline of areca nut consumed 1 day before the blood was drawn remained at about 7 ng/mL [46]. Indeed, the pathophysiology of cardiac fibrosis is highly complicated, as elements of cardiac fibrosis are involved in a wide range of multifactorial disorders. This study has certain limitations. First, the expression trends of some proteins were not consistent in rats that received low and high doses; thus, we need more doses to validate the effects of arecoline in cardiac fibrosis. Second, although our results showed that cardiac fibrosis is attributed to the activation of the TGF-β/Smad pathway, whether or not other signaling molecules are involved in arecoline-induced cardiac fibrosis remains to be elucidated. Third, there is still a small number of repetitions and that additional research is necessary for confirmation.

## 5 Conclusion

Unlike the quick decline of arecoline in our animal model, the plasma concentration of arecoline in habitual areca nut chewers remains substantial, which may lead to more profoundly harmful effects. Therefore, the use of areca nuts must be tightly regulated for the welfare of society.
